# Factors associated to clinical learning in nursing students in primary health care: an analytical cross-sectional study

**DOI:** 10.1590/1518-8345.0327.2803

**Published:** 2016-09-09

**Authors:** Pilar Serrano-Gallardo, Mercedes Martínez-Marcos, Flora Espejo-Matorrales, Tiemi Arakawa, Gabriela Tavares Magnabosco, Ione Carvalho Pinto

**Affiliations:** 1 PhD, Professor, Departamento de Enfermería, Universidade Autonoma de Madrid, Madrid, Spain. Researcher, Research Institute for Higher Education and Science, Health Research Institute Puerta de Hierro, Madrid, Spain.; 2 MSc, RN, Southeastern Primary Health Care Area, Ayuntamiento de Madrid, Madrid, Spain.; 3 Post-doctoral fellow, Escola de Enfermagem de Ribeirão Preto, Universidade de São Paulo, PAHO/WHO Collaborating Centre for Nursing Research Development, Ribeirão Preto, SP, Brazil. Scholarship holder from Fundação de Amparo à Pesquisa do Estado de São Paulo (FAPESP), Brazil.; 4 PhD, Associate Professor, Escola de Enfermagem de Ribeirão Preto, Universidade de São Paulo, PAHO/WHO Collaborating Centre for Nursing Research Development, Ribeirão Preto, SP, Brazil.

**Keywords:** Nursing Education Research, Primary Health Care, Students, Nursing, Mentors

## Abstract

**Objective:**

to identify the students' perception about the quality of clinical placements and asses the influence of the different tutoring processes in clinical learning.

**Methods:**

analytical cross-sectional study on second and third year nursing students (n=122) about clinical learning in primary health care. The Clinical Placement Evaluation Tool and a synthetic index of attitudes and skills were computed to give scores to the clinical learning (scale 0-10). Univariate, bivariate and multivariate (multiple linear regression) analyses were performed.

**Results:**

the response rate was 91.8%. The most commonly identified tutoring process was "preceptor-professor" (45.2%). The clinical placement was assessed as "optimal" by 55.1%, relationship with team-preceptor was considered good by 80.4% of the cases and the average grade for clinical learning was 7.89. The multiple linear regression model with more explanatory capacity included the variables "Academic year" (beta coefficient = 1.042 for third-year students), "Primary Health Care Area (PHC)" (beta coefficient = 0.308 for Area B) and "Clinical placement perception" (beta coefficient = - 0.204 for a suboptimal perception).

**Conclusions:**

timeframe within the academic program, location and clinical placement perception were associated with students' clinical learning. Students' perceptions of setting quality were positive and a good team-preceptor relationship is a matter of relevance.

## Introduction

The European Union (EU) policy on nursing education has been changing procedures in nursing schools around Europe, aiming to unify the educational structure and guarantee equality in professional qualification. According to EU recommendations, at least 50% of the total hours from nursing studies has to be completed with clinical practicum experiences (77/453/CEE), and students must be supervised by a professional nurse in these practice sessions(1).

New learning models also emphasize the importance of practicum settings with the purpose of achieving an adequate competence development by the student(2). The clinical placement, or clinical location, has been defined as the interactive network of forces within the clinic that has an influence on the clinical results of students' learning(3). It is known that not every clinical placement can provide nursing students with a positive learning environment(4), and, considering that students spend a significant part of their training in these settings, an evaluation of this scenario and the feedback of students about the quality of their learning, should be a priority for those involved with nursing education(5). 

The literature shows that the quality of the learning environment is dependent on a variety of factors, including characteristics of clinical placement, the degree of compatibility to the learning objectives and the capacity to provide opportunities for students to learn, as well as the relationship among students, health professionals and university faculty(6). The feeling of recognition/attachment in the clinical learning placements and an authentic relationship of students with the tutors and health team members are considered as key elements to stimulate students´ self-confidence and reliability, which favors the learning process(7).

Factors that students identify as learning facilitators include the promotion of responsibility and autonomy, provision of opportunities to perform different tasks, provision of support, as well as feedback of students´ performance from preceptors and professors(8). Variables considered to hinder the learning process include lack of trust in nursing students shown by preceptors, discontinuity in supervision, scarcity of opportunities to perform practical procedures, and feelings of inadequacy and low self-confidence among students(9). 

The students' perceptions about the learning setting quality and the tutoring model can provide valuable information to educators related to the learning process in the clinical practicum environment. However, it should be pointed out that few assessment tools have been developed to investigate such perceptions(10). In addition, tutoring models can influence the learning process within clinical placement. Among the many different tutoring model definitions found in the literature, the preceptorship model, in which a student is under the supervision of a registered nurse, is one of the most frequent for nursing education(11). The outcomes of the tutoring models for clinical learning are also an issue that needs further investigation, especially when it come to Primary Health Care (PHC) practicum experiences(12) . 

The aim of the present study was to assess students´ perceptions on the quality of clinical placements in PHC and to evaluate the influence of different tutoring processes on student learning. 

## Methods

A cross-sectional analytical study was conducted with 122 students in the 2^nd^ and 3^rd^ year of their nursing degree from *Puerta de Hierro* School (Autonomous University of Madrid, Madrid, Autonomous Community of Madrid, Spain) during the academic period of 2009 and 2010. 

Clinical learning was conducted for 5 weeks in PHC services from three health areas inside the autonomous region of Madrid. In parallel with their clinical learning, students had to attend two subjects on Community Nursing, which were offered during both the second year and third year of the nursing degree. Students could choose to do clinical learning during any of the three periods of an academic year. 

Each student had a preceptor who was responsible for his/her supervision during clinical learning. The preceptor was a registered nurse working in PHC services, who "assumed voluntarily the responsibility of clinical and practical learning of students within his/her working place during his/her working hours; by planning, coordinating and evaluating the learning process"^13^. 

Besides the preceptor, the professor was also involved with the clinical practicum experience. The professor was a faculty member who coordinated and supervised the clinical learning process in its entirety, ensured communication between student and preceptor, and acted as a learning facilitator. 

A synthetic Score^14^, from 0 to 10, was calculated to grade the clinical learning. By using a structured questionnaire, preceptors evaluated students' attitudes and skills in the clinical placement, during home visits and related to nursing procedures (comprising 40% of the synthetic score) and the student conducted a self-assessment (comprising 15% of the synthetic score). Two written assignments were graded by the responsible professor, one focused on a clinic case (25% of the score) and the other focused on a health situation analysis in the health area (20% of the score). This synthetic score was applied and validated in a previous study^14^. We also considered the final grade of "Community Nursing" subjects, which consisted of a written test scored from 0 to 10 to describe students' performance. 

The tutoring model was defined as the supportive process provided during clinical learning, characterized by evaluation meetings, the use of active teaching strategies, and active communication among students, preceptors and professors^2^. In order to define an operational definition of tutoring model, we used a structured observational guide to verify three kinds of tutoring process: 

-Student-professor process: communication during clinical learning period by email and/or submission/feedback of drafts of assignments mentioned above.

-Preceptor-professor process: communication during clinical learning period by email and/or having attended the final evaluation meeting.

-Mixed process: the two above-mentioned tutoring processes occurred. 

**Figure 1:**
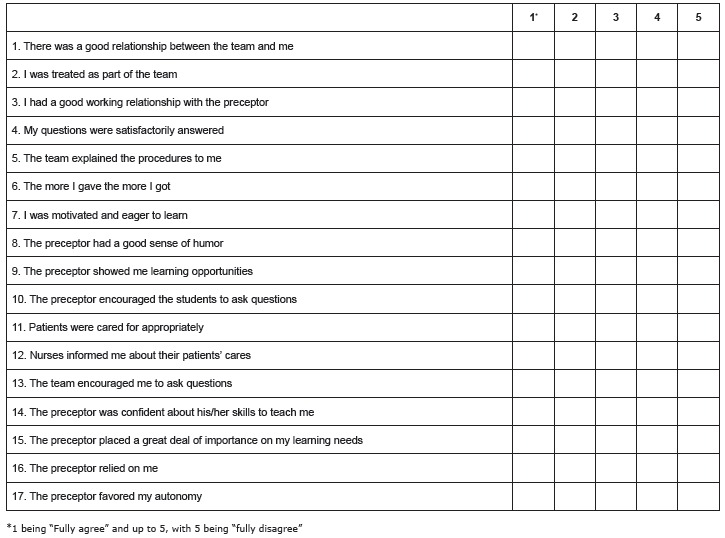
Modified version of the Clinical Placement Evaluation Tool

The student's perception of the quality of the clinical placement was assessed by a modified version of the Clinical Placement Evaluation Tool (CPET), which consisted of a self-administered questionnaire of 17 items with a five-point Likert scale (Figure 1). After having permission from its authors (Mosely, Mead and Moran from the University of Glamorgan, United Kingdom), the original tool was adapted and validated ^14^ for Spanish language and culture; presenting a Cronbach's alpha value of 0.89 ^15^. In this CPET version, a lower score means a better setting perception. An optimal perception of the clinical placement was considered for those scores below the 50th percentile value, and a suboptimal perception was considered for those scores above the 50th percentile value^16^. 

The CPET questionnaire was provided to students at the last day of the clinical learning period. Students were oriented to fill out the questionnaire within 48 hours and delivered it to the professor who coordinated the clinical learning.

The clinical learning grade (measured on a 0 to 10 scale) - obtained by the synthetic score as described above - was considered as the dependent variable. 

The independent variables were: the students´ perception of the quality of the clinical placement - as obtained by the modified CPET version; and the types of tutoring process (professor-student, professor-preceptor, mixed). Student's age; student´s sex; the clinical placement location (PHC Area, named with letters A, B or C), the academic year (second or third year), the academic period (1^st^, 2^nd^ or 3^rd)^ and final grade of the "Community Nursing" subjects were also included as independent variables.

For data analysis, we performed univariate analyses (measures of central tendency and dispersion or percentages, depending on the variables' nature) and bivariate analyses (Student's t-test, ANOVA, and Pearson correlation coefficient). A multivariable analysis was also developed using a multiple linear regression. The dependent variable was clinical learning and the explanatory variables were those aforementioned as independent variables, which were associated with clinical learning at a bivariate level, considering a p-value of ≤ 0.20. A significance level of less than 0.05 was used for all analyses (except for regression analysis). The dummy variables were considered significant even if some categories had not presented a p-value of £ 0.05^17^. Confidence intervals (95%) were estimated. The SPSS v.17 software was used. 

The study was conducted according to the ethical guidelines of the Declaration of Helsinki. To carry out the study, institutional permissions were obtained. The study objectives and procedures were previously explained to the students and ethical procedures with data management were followed strictly. As students may be considered a vulnerable population, the participation was voluntary and informed consent was obtained from all subjects who agreed to participate. 

## Results

The response rate was 91.8% (n=112). The average age of students was 22.06 years with a standard deviation (SD) of 4.7 years. The majority of respondents were women (91.9%, n=109). A total of 56.3% (n=63) were third-year students and 42.3% (n=47) carried out their clinical learning in the PHC Area B. In regard to the tutoring process, 45.2% of students (n=42) identified a professor-preceptor process, 29% (n=27) identified a mixed process and only 10.8% (n=10) identified a professor-student process. Data on tutoring process were not available for 15.1% (n=14) of students. The clinical placement was assessed as "optimal" by 55.1% (n=59) of students. The average of Clinical Learning grade was 7.89 (SD= 0.84; CI95%: 7.73-8.06). The average grade of the Community Nursing subjects was 6.52 (SD= 1.49; CI95%: 6.24-6.80) (Table 1).

**Table 1:** Description of the studied population. Madrid. Spain, 2009-2010

		Mean	SD^*^	CI95%^†^
Student´s age (in year)		22.06	4,7	[21.16, 22.99]
CPET^‡^ summary score		26.25	14.28	[23.51, 28.99]
Mean grade score for Clinical Learning		7.89	0.84	[7.73, 8.06]
Mean grade score for Community Nursing subjects		6.52	1.49	[6.24, 6.80]
		n	%	
Student´s sex:	Female	102	91.9	
	Male	9	8.1	
Academic year:	Second	49	43.7	
	Third	63	56.3	
Clinical placement location^||^:	PHC^§^ Area A	25	22.5	
	PHC^§^ Area B	47	42.3	
	PHC^§^ Area C	39	35.2	
Academic period:	1^st^	38	33.9	
	2^nd^	34	30.4	
	3^rd^	40	35.7	
Tutoring process:	Mixed process	27	29.0	
	Professor-student process	10	10.8	
	Preceptor-professor process	42	45.2	
	No data was obtained	14	15.1	
Clinical Placement perception^¶^:	Optimal	59	55.1	
	Suboptimal	48	44.9	

*SD: Standard Deviation; †CI95%: Confidence Intervals (95%); ‡CPET: Clinical Placement Evaluation Tool; §PHC: Primary Health Care;

||Valid responses total number are 111; ¶Valid responses total number are 107.

**Table 2:** Description of the modified version of the Clinical Placement Evaluation Tool Madrid. Spain, 2009-2010

	Maximum agreement								Minimum agreement	
	(1)		(2)		(3)		(4)		(5)	
	%	(n)	%	(n)	%	(n)	%	(n)	%	(n)
Team: good relationship	80.4	(90)	10.7	(12)	3.6	(4)	2.7	(3)	2.7	(3)
Treated like a member	68.8	(77)	20.5	(23)	4.5	(5)	2.7	(3)	3.6	(4)
Preceptor: good relationship	77.7	(87)	13.4	(15)	2.7	(3)	2.7	(3)	3.6	(4)
Answered questions	68.8	(77)	23.3	(25)	3.6	(4)	1.8	(2)	3.6	(4)
Team: explained	52.7	(59)	32.1	(36)	8	(9)	0.9	(1)	6.3	(7)
I gave - I got*	61.3	(68)	25.2	(28)	7.2	(8)	2.7	(3)	3.6	(4)
Motivated and eager*	77.5	(86)	11.7	(13)	6.3	(7)	0.9	(1)	3.6	(4)
Preceptor: sense of humor	69.6	(78)	17.9	(20)	7.1	(8)	0.9	(1)	4.5	(5)
Preceptor: opportunities	71.4	(80)	17.9	(20)	5.4	(6)	0.9	(1)	4.5	(5)
Preceptor: encouraged me to ask*	60.4	(67)	25.2	(28)	5.4	(6)	3.6)	(4)	5.4	(6)
Patient: good care^†^	64.5	(71)	22.7	(25)	8.2	(9)	0	(0)	4.5	(5)
Care information	52.7	(59)	36.6	(41)	3.6	(4)	2.7	(3)	4.5	(5)
Team: encouraged me to ask*	38.4	(43)	38.4	(43)	13.5	(15)	7.2	(8)	1.8	(2)
Confident preceptor	74.1	(83)	17.9	(20)	2.7	(3)	0.9	(1)	4.5	(5)
Preceptor: learning importance	73.2	(82)	15.2	(17)	5.4	(6)	2.7	(3)	3.6	(4)
Preceptor: reliance on me	73.2	(82)	16.1	(18)	3.6	(4)	1.8	(2)	5.4	(6)
Preceptor: favors my autonomy	67.9	(76)	17.9	(20)	5.4	(6)	3.6	(4)	5.4	(6)

*Valid responses total number are 111; †Valid responses total number are 110.

Regarding the CPET items, a higher level of agreement was identified in the following items: "There was a good relationship between the team and me" (80.4%; n=90), "I had a good working relationship with the preceptor" (77.7%; n=87) and "I was motivated and eager to learn" (77.5%; n=86). A lower level of agreement was identified in the items: "The team explained the procedures to me" (52.7%; n=59), "Nurses informed me about their patients' cares" (52.7%; n=59), and "The team encouraged me to ask questions" (38.4%; n=43) (Table 2).

The highest grades in clinical learning in the bivariate analysis are related to women, third-year students, PHC Area B, tutoring process "professor-student" and clinical placement perceived as optimal. However, statistical significance was found only for the "academic year" (7.17 in second year and 8.36 in third year, p<.001) and "PHC Area" (7.44 in area A; 8.01 in area B and 7.86 in area C, p=0.03) (Table 3). 

**Table 3:** Mean score and Confidence Interval (95%) for "Clinical Learning" according to the study variables. Madrid. Spain, 2009-2010

		Mean	[CI95%]*	p value
Sex	Female	7.88	[7.72, 8.06]	0.081
	Male	7.35	[6.55, 8.16]	
Academic year	Second	7.17	[7, 7.35]	<.001
	Third	8.36	[8.2, 8.54]	
Clinical placement location	PHC^†^ Area A	7.44	[7.05, 7.84]	0.03
	PHC^†^ Area B	8.01	[7.79, 8.25]	
	PHC^†^ Area C	7.86	[7.87, 8.15]	
Academic period	1^st^	7.78	[7.53, 8.04]	0.884
	2^nd^	7.86	[7.52, 8.2]	
	3^rd^	7.88	[7.59, 8.17]	
Tutoring process	Mixed	7.98	[7.63, 8.34]	0.275
	Professor-Student	8.48	[8.03, 8.92]	
	Professor-preceptor	7.75	[7.49, 8.02]	
	No data of tutoring process	7.81	[7.26, 8.37]	
Clinical Placement perception	Optimal	7.98	[7.76, 8.22]	0.061
	Suboptimal	7.66	[7.41, 7.92]	

* CI95%: Confidence Intervals (95%)

† PHC: Primary Health Care

There was no evidence of an association between the clinical learning and students' age (Pearson correlation coefficient: 0.22; p=0.820). However, there was evidence for an association with the grades obtained in the "Community Nursing" subjects (Pearson correlation coefficient: 0.435; p<001).

The multiple linear regression model (adjusted for age, sex and grades in the "Community Nursing" subjects) presents a good explanatory capacity (coefficient of determination= 0.597; F=19.459, p<.001). It included the variables "academic year" (beta coefficient = 1.042 for third year, reference category being the second year), "PHC Area" (beta coefficient = 0.271 for area number C and beta coefficient = 0.308 for area B, reference category being area A) and "clinical placement perception" (beta coefficient = - 0.204 for suboptimal perception where optimal perception is the reference category) (Table 4).

**Table 4:** Multiple linear regression model for the dependent variable "Clinical Learning". Madrid. Spain, 2009-2010

	Beta Coefficient	t	p value	CI95% for Beta - Lower limit	CI95% for Beta - Upper limit
Constant	4.523	8.953	<.001	3.519	5.526
Age	-.004	-.305	.761	-.027	.020
Third year*	1.042	8.796	<.001	.806	1.277
Sex^†^	-.243	-.995	.322	-.728	.242
Suboptimal Clinical Placement perception^‡^	-.204	-1.750	.083	-.435	.027
PHC Area B^§^	.308	2.116	.037	.019	.598
PHC Area C^§^	.271	1.801	.075	-.028	.570
Grade in the Community Nursing subjects	.140	3.474	.001	.060	.221

Coefficient of determination= 0.597; F=19.459; p=0.000

* "Second year" is the reference category

†"Female" is the reference category

‡"Positive Clinical Placement perception" is the reference category

§"HC" (Primary Health Care) Area A" is the reference category

## Discussion 

Our findings highlight that the clinical learning of nursing students in PHC settings is associated with the particular timeframe within their degree studies (better results in the third year), with the clinical learning location, and with the perception about the clinical placement (better grades when there is an optimal perception). A qualitative study with a phenomenological approach^18^ shows the importance that students attribute to clinical placement in order to achieve good clinical learning experiences. Moreover, they point out that the health professionals have a big influence on the student, who needs to receive recognition and support from the different members of the team, apart from his/her preceptor. Other studies also confirm that the relationship between students and clinical nurses has a significant influence on the learning experiences in clinical placement^19^ ^-^ ^20^. Other authors have noted that communication and cooperation are the basis of adequate supervisory relationships^11^. Further, Bisholt et al.^21^, concluded that having meaningful learning situations was a relevant aspect highlighted by students. These results are consistent with those obtained in the present study, where the elements that were best perceived by students focused on the good relationship either with the team or with the preceptor and on the motivation to learn. 

Higher clinical learning showed by third-year students could be explained by the fact that those students had been using a reflective methodology based on self-assessment for two years (during the second and third year) as opposed to second-year students who had only used it for a year. This coincides with the conclusions obtained in a literature review^22^ showing that reflective activities provide opportunities to students for critical thinking development and tools for self-learning. In addition, Brugnolli and colleagues point out that an effective preceptorship is the one that includes a reflective work process, highlighting the active role of students guiding their own learning process^23^. 

Regarding the PHC Area where clinical learning was performed, this study does not allow us to clarify why the area influences clinical learning in a relevant and independent way. It is important to mention that there were no significant differences among those PHC Areas included in the study related to the academic year, the tutoring process they performed, and the student perception about the clinical placement they had. Some other factors described in the literature but not included in this research, such as an appropriate training for preceptors, the pedagogical atmosphere and effective leadership patterns, may possibly play a role to explain this influence^11^. 

Tutoring processes linking professors, preceptors and students, while having influence on learning, did not remain as an explanatory variable in the multivariable models. However, it is important to emphasize a study that showed that two of the six identified essential factors for a good quality clinical learning setting are: intrinsic student motivation for his/her self-management and control that students may have to design their own learning^24^. A pragmatic clinical trial analyzed the impact that tutoring strategies have on the accuracy of diagnostic reasoning; it demonstrated that the experimental group of students made less incorrect hypotheses in simulated cases^25^. Another quasi-experimental study^26^ highlighted that the teaching portfolio (which included reflexive dynamics and self-assessment) improved students' clinical skills, especially in performance of case reports. The clinical supervision conducted by professors fosters more challenging behaviors: students discuss more of their learning needs, establish more connections between theory and practice, and are more motivated for reflection^27^. These findings are consistent with our results, showing that students with better grades in clinical learning were those who had an active role in the tutoring process and sent emails and drafts of their assignments to the professor (tutoring process professor-student). Morley^28^ shows that supporting student nurses in practice with additional online communication tools is an effective mechanism to improve clinical learning.

On the other hand, preceptorship strategies that stimulate students to raise questions and go deeper in knowledge construction, are considered by the students as being more effective^23^, and these aspects presented bigger deficiencies in the clinical placements analyzed in this study. These findings may bring elements to help understand the lower grades in clinical learning when the tutoring process was preceptor-professor, in which the student did not participate in the process. 

In general, the students' perception about quality in clinical placement in PHC is highly positive, as also shown by other studies^11^ ^,^ ^29^. Placement in PHC indicate a higher range of opportunities to learn from preceptors who organize and plan the clinical learning, apart from having a closer personal relationship with the student. 

Regarding the study's limitations, it is worth mentioning that the sample size did not allow us to conducts stratified or subgroup analyses, which would have been relevant to further research into the tutoring process (there were too few individuals in some of the categories). In addition, a potential information bias related to socially desirable responses needs to be considered, even with the fulfillment of ethical procedures, as this may contribute to an overestimation of the positive assessment of learning environments.

As strengths of this study, it should be noted the high rate of response achieved; which reduces the likelihood of selection bias. Furthermore, the analytical character of the design ensuring the appropriate time sequence between the influencing factors and the outcome variable, contributes to an important criteria for causality. Moreover, the CPET is a tool that has allowed us to obtain reliable and valid data after its adaptation and validation to the Spanish environment.

Based on the scope of the study, generalizability of findings would be limited to PHC clinical learning environments. Further research is needed to explore these relationships in other types of clinical placements. We can say that the findings have external validity for all those clinical learning environments in undergraduate nursing education in which each student is assigned to a nurse preceptor, in addition to a professor responsible for the clinical learning.

## Conclusions

The students' clinical learning in PHC is associated with the timeframe within the degree program, the location where it is carried out and the clinical placement perception. A good relationship, including feedback and reflective learning strategies between preceptor and student, is very important for the development of an adequate educational setting oriented to optimum clinical learning. In general, students' perceptions about the quality of practice settings are highly positive, and PHC is known as a field that provides good opportunities for students to improve their competences and skills. 

The analysis of clinical placements shows the essential elements for students to learn. Those elements allow the appropriate design of clinical learning in professional settings and the development of competences for future professional nurses. 

Learning to be a nurse is a multidimensional process that demands time from two different perspectives: nursing practice in the field, and a relationship of supervision and support in adequate learning settings. The students' clinical perspective on quality of education contributes to the knowledge for the development of better educational experiences. 
